# Calculation of left ventricular volumes and ejection fraction from dynamic cardiac-gated ^15^O-water PET/CT: 5D-PET

**DOI:** 10.1186/s40658-017-0195-2

**Published:** 2017-11-14

**Authors:** Jonny Nordström, Tanja Kero, Hendrik Johannes Harms, Charles Widström, Frank A. Flachskampf, Jens Sörensen, Mark Lubberink

**Affiliations:** 1 Nuclear Medicine and PET, Department of Surgical Sciences, Uppsala University, SE-751 85 Uppsala, Sweden; 2 Centre for Research and Development, Uppsala University, Gävle, Gävleborg County Sweden; 3 Medical Imaging Centre, Uppsala University Hospital, Uppsala, Sweden; 4 Cardiology, Department of Medical Sciences, Uppsala University, Uppsala, Sweden; 5 Medical Physics, Uppsala University Hospital, Uppsala, Sweden; 6 Department of Nuclear Medicine and PET Centre, Aarhus University Hospital, Aarhus, Denmark

## Abstract

**Background:**

Quantitative measurement of myocardial blood flow (MBF) is of increasing interest in the clinical assessment of patients with suspected coronary artery disease (CAD). ^15^O-water positron emission tomography (PET) is considered the gold standard for non-invasive MBF measurements. However, calculation of left ventricular (LV) volumes and ejection fraction (EF) is not possible from standard ^15^O-water uptake images. The purpose of the present work was to investigate the possibility of calculating LV volumes and LVEF from cardiac-gated parametric blood volume (*V*_B_) ^15^O-water images and from first pass (FP) images.

Sixteen patients with mitral or aortic regurgitation underwent an eight-gate dynamic cardiac-gated ^15^O-water PET/CT scan and cardiac MRI. *V*_B_ and FP images were generated for each gate. Calculations of end-systolic volume (ESV), end-diastolic volume (EDV), stroke volume (SV) and LVEF were performed with automatic segmentation of *V*_B_ and FP images, using commercially available software. LV volumes and LVEF were calculated with surface-, count-, and volume-based methods, and the results were compared with gold standard MRI.

**Results:**

Using *V*_B_ images, high correlations between PET and MRI ESV (*r* = 0.89, *p* < 0.001), EDV (*r* = 0.85, *p* < 0.001), SV (*r* = 0.74, *p* = 0.006) and LVEF (*r* = 0.72, *p* = 0.008) were found for the volume-based method. Correlations for FP images were slightly, but not significantly, lower than those for *V*_B_ images when compared to MRI. Surface- and count-based methods showed no significant difference compared with the volume-based correlations with MRI. The volume-based method showed the best agreement with MRI with no significant difference on average for EDV and LVEF but with an overestimation of values for ESV (14%, *p* = 0.005) and SV (18%, *p* = 0.004) when using *V*_B_ images. Using FP images, none of the parameters showed a significant difference from MRI. Inter-operator repeatability was excellent for all parameters (ICC > 0.86, *p* < 0.001).

**Conclusion:**

Calculation of LV volumes and LVEF from dynamic ^15^O-water PET is feasible and shows good correlation with MRI. However, the analysis method is laborious, and future work is needed for more automation to make the method more easily applicable in a clinical setting.

## Background

Quantitative measurement of myocardial blood flow (MBF) is of increasing interest in the clinical assessment of patients with suspected coronary artery disease (CAD). Several previous studies have stressed the added value of quantification of MBF over qualitative perfusion imaging [1–4]. Dynamic positron emission tomography (PET) can be used to measure MBF using various tracers such as ^13^N-amonia, ^82^Rb or ^15^O-water [5–7]. ^15^O-water is generally considered to be the gold standard for non-invasive measurement of MBF since it is freely diffusible and metabolically inert. Changes in myocardial ^15^O-water activity are solely dependent on MBF and are not affected by disease-specific/dependent variations in extraction fraction or metabolic interactions. However, since water is freely diffusible, static uptake images provide no information on MBF. MBF is therefore usually calculated using tracer kinetic modelling on dynamic scan data on a voxel-by-voxel basis, resulting in parametric images showing MBF at the voxel level [4, 8].

In addition to MBF, assessments of cardiac function and left ventricular (LV) volumes hold important diagnostic and prognostic information [9, 10]. PET tracers with high retention in the myocardium lend themselves to ECG-gated assessments of LV volumes and LV ejection fraction (EF) and can thus be used for routine diagnosis of LV dysfunction [11–13]. ^15^O-water, on the other hand, has low contrast between tracer concentration in the myocardium and the blood. This has traditionally ruled out ECG-gated ^15^O-water PET for measuring EF. Consequently, LV volumes have to be calculated using, for instance, echocardiography or MRI, which requires a prolonged or additional hospital visit. Another option could be to use ^15^O-water cardiac-gated images acquired during the first pass (FP), which has been shown a feasible method to extract cardiac functional data [14]. The purpose of the present work was to investigate the possibility of calculating LV volumes and LVEF from a dynamic ^15^O-water PET/CT using cardiac-gated parametric blood volume images and to compare this to FP-based values.

## Methods

### Patient characteristics

Data from a total of 16 patients were used in this pilot study (12 men and 4 women, age range 35–81, mean age 58). The patients were consecutively included from a larger study on mitral or aortic regurgitation, no selection was done. All had minimal symptoms of heart failure (NYHA class I–II), and none had any documented history of CAD. The regional ethical committee approved the project, and all patients gave their written informed consent prior to inclusion in the study.

### Image acquisition

^15^O-water scans were acquired using a Discovery ST PET/CT scanner (GE Healthcare, Waukesha, MI). First, a respiration-averaged low-dose CT scan (10 mA, 1 s rotation time, 0.562 pitch, full helical) during normal breathing was acquired for attenuation correction. Then, 400 MBq of ^15^O-water was administrated intravenously as a fast bolus (5 mL at 1 mL/s, followed by 35 mL saline at 2 mL/s) during rest, simultaneously with the start of a 6-min 3D cardiac-gated list mode emission scan with ECG information acquired in parallel to the list mode data. Images were reconstructed into eight gates with 20 frames per gate (1 × 10, 8 × 5, 4 × 10, 2 × 15, 3 × 20 and 2 × 30 s) using ordered subset expectation maximisation (OSEM) with two iterations and 21 subsets, and a 5-mm Gaussian post-filter. Image dimensions were 128 × 128 × 47 with a voxel size of 3.9 × 3.9 × 3.27 mm.

Two to 4 h after the ^15^O-water scans, MRI scans were performed on a 3-T MRI scanner (Achieva, Philips Healthcare) with an 80 mT/m gradient system, using a 32-channel cardiac coil in supine position and retrospectively gated vector ECG for cardiac triggering. Functional images were obtained with a single-shot steady-state free precession (SSFP) cine sequence covering the left ventricular myocardium from apex to base in 6-mm-thick short-axis slices with 4-mm gaps. The following parameters were used: TR shortest (3.4 ms), TE shortest (1.7 ms), flip angle 45°, bandwidth 1243 Hz/pixel, 30 phases/cardiac cycle, field-of-view 320 mm and matrix of 160 × 154.

### Parametric images

The basis function implementation [15, 16] of the single tissue compartment model with corrections for spill-over and partial volume effects [17, 18] was used to generate parametric images for each gate:

1 $$ {C}_T(t)=\mathrm{PTF}\cdot \mathrm{MBF}\cdot {C}_{\mathrm{A}}(t)\kern0.5em \otimes \kern0.5em {e}^{-\frac{\mathrm{MBF}}{V_{\mathrm{T}}}\cdot t}+{V}_{\mathrm{A}}\cdot {C}_{\mathrm{A}}(t)+{V}_{\mathrm{RV}}\cdot {C}_{\mathrm{RV}}(t) $$

where *V*_A_ represents arterial blood volume and left ventricular spill-over fraction, *V*_RV_ venous blood volume and right ventricular spill-over fraction, PTF perfusable tissue fraction and *V*_T_ the partition coefficient, which was fixed to 0.91 mL g^−1^ [19]. A set of 50 basis functions was precomputed using logarithmically spaced values of MBF_i_ between 0.1 and 2.2 mL g^−1^ min^−1^:

2 $$ {B}_{\mathrm{i}}(t)={\mathrm{MBF}}_{\mathrm{i}}\cdot {C}_{\mathrm{A}}(t)\otimes {e}^{-\frac{{\mathrm{MBF}}_{\mathrm{i}}}{V_{\mathrm{T}}}\cdot t} $$

The linear combination of *B*_i_(*t*), *C*_A_(*t*) and *C*_RV_(*t*) that minimised the sum of square differences with *C*_T_(*t*), gave PTF, *V*_A_, *V*_RV_ and MBF. For each scan, a single set of input functions, *C*_A_(*t*) and *C*_RV_(*t*), was generated from non-gated data using cluster analysis in Cardiac VUer [8]. This set was then applied to all gated images to generate gated parametric images using in-house developed software in Matlab 12, based on the same scripts as Cardiac VUer. Parametric blood volume images (*V*_B_) were generated as the sum of *V*_A_ and *V*_RV_ images.

### First pass images

Standard FP uptake images of the left and right ventricles were generated in Matlab 12 by summation of frames ranging from 10 to 50 s after injection. For two patients with low cardiac output, frames between 20 and 70 s were used.

### LVEF calculations

From ^15^O-water, LV volumes and LVEF were calculated from *V*_B_ and FP images using blood-pool gated SPECT (BPGS), a program based on Myovation (GE Healthcare) and QBS (Cedars Sinai). Gated transaxial *V*_B_ and FP images were imported into the application on a Xeleris workstation for automatic segmentation and quantification. QBS provides three different methods for calculation of LV volumes and LVEF: surface- (s), count- (c) and volume-based (v) methods. The results from all three methods were used for comparison.

From MRI, LV volumes and LVEF were calculated using ViewForum (Philips). The endocardial contour was manually traced in end-diastole and in end-systole. The most basal slices had to show at least 50% visible myocardial circumference to be included. The LV outflow tract was included in the endocardial contour using the aortic valve as the lateral border. The papillary muscles and trabeculae were included in the blood volume. Stroke volume (SV) was calculated as EDV-ESV and LVEF as SV/EDV expressed as a percentage. All ^15^O-water-based LV volumes and LVEF calculations were performed by a medical physicist (JN), and for assessment of inter-operator repeatability, calculations were also performed by an experienced nuclear medicine physician (TK).

### Statistics

Test for normality was done using a Shapiro-Wilk test, and correlations between ^15^O-water-based and MRI-based end-systolic volumes (ESV), end-diastolic volumes (EDV) and LVEF were assessed using Pearson’s *r*. The agreement was assessed using Bland Altman analysis and differences of means using Student’s paired *t* test. Inter-operator repeatability was assessed using intraclass correlation coefficient (ICC).

## Results

**Fig. 1:**
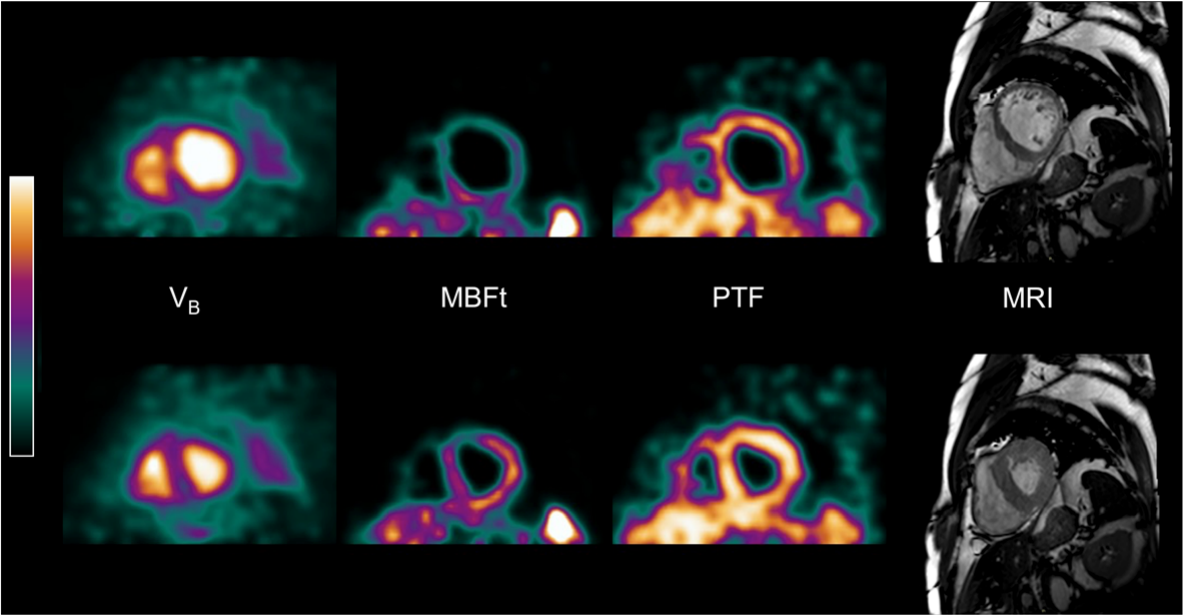
End-diastolic (top row) and end-systolic (bottom row) short-axis parametric ^15^O-water blood volume (*V*_B_), transmural blood flow (MBFt) and perfusable tissue fraction (PTF) images, as well as MRI images of a typical patient. Colour scales for end-diastolic and end-systolic images are identical

**Fig. 2:**
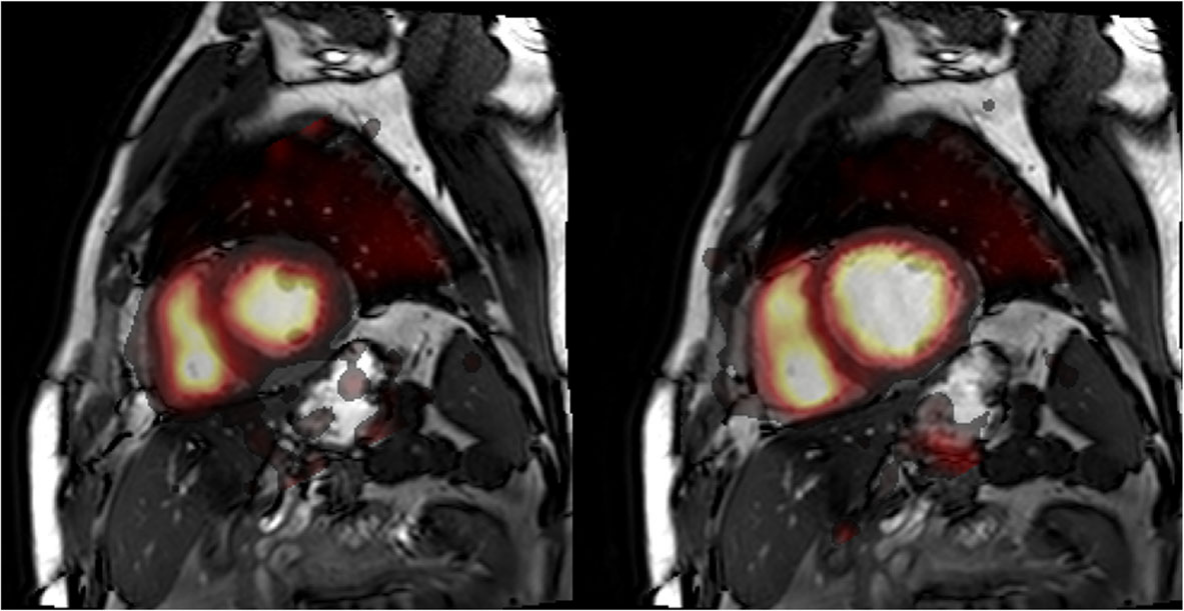
End-diastolic (left) and end-systolic (right) fusion between parametric ^15^O-water blood volume (*V*_B_) and MRI images

**Fig. 3:**
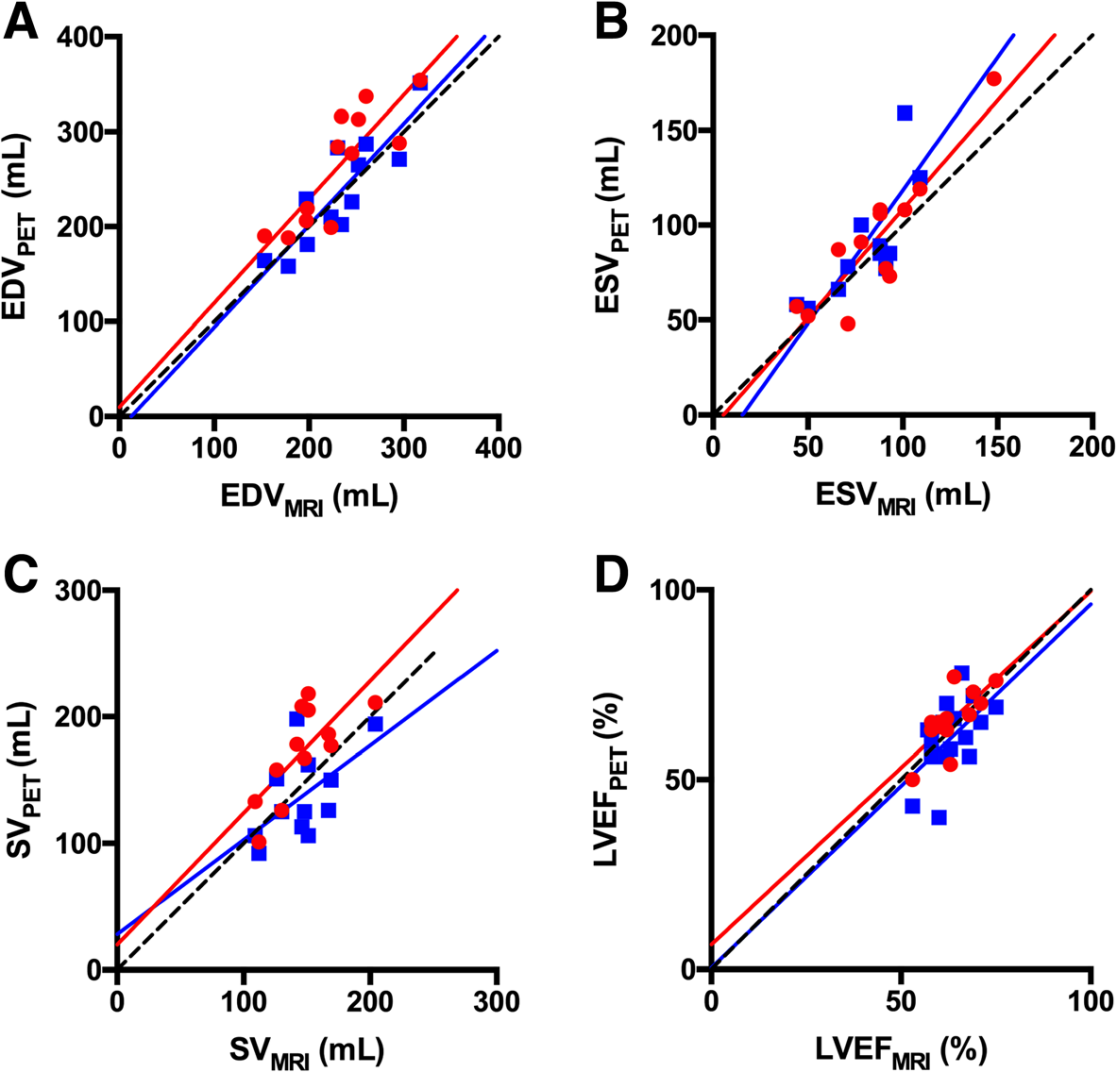
Correlation between ^15^O-water PET volume-based method and MRI ESV (**a**), EDV (**b**), SV (**c**) and LVEF (**d**). Circles (red) are values from *V*_B_ images, and squares (blue) are values from FP images. Solid lines are linear regression lines, and dashed lines are lines of identity. Outliers were removed

**Fig. 4:**
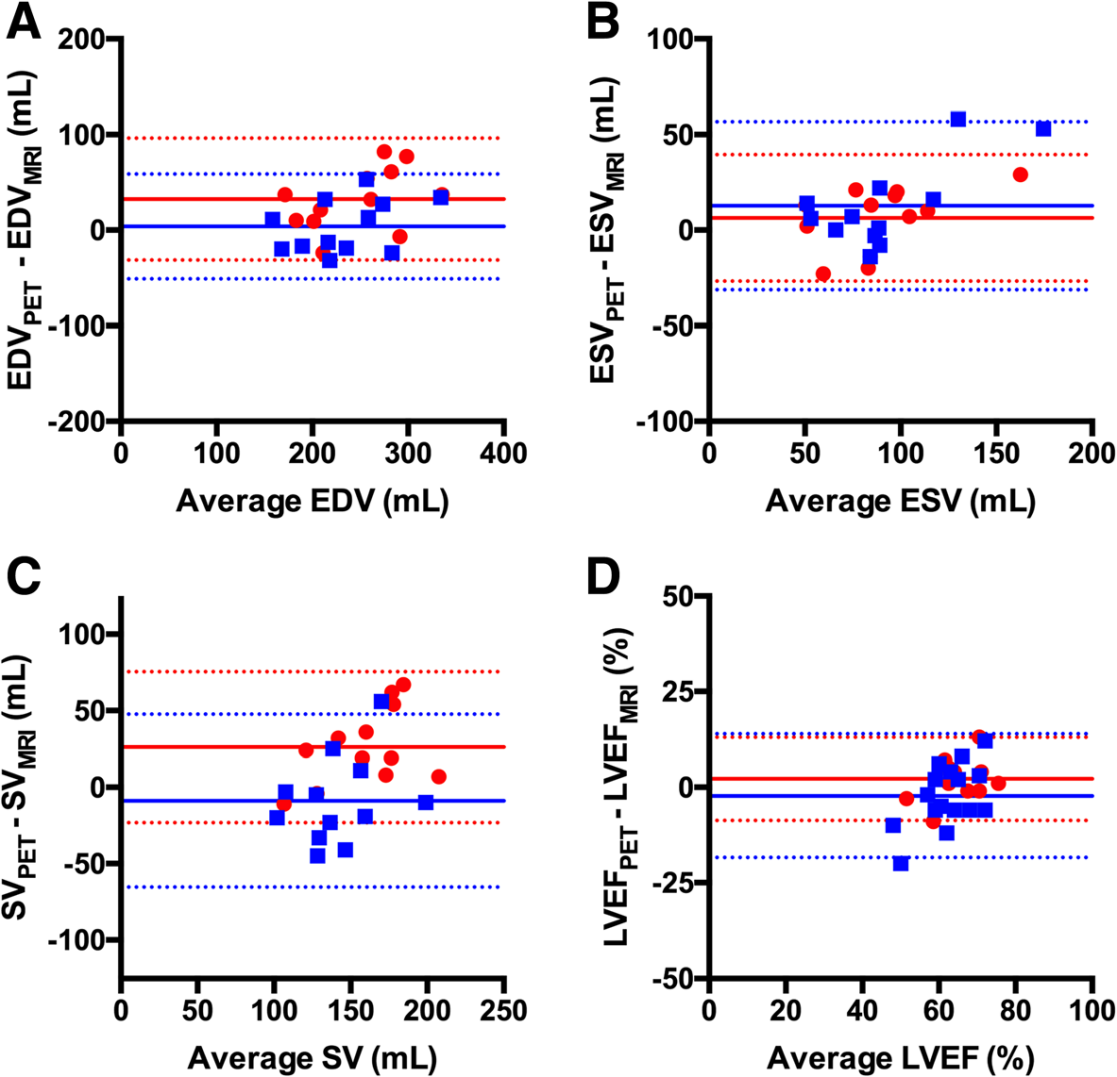
Bland Altman plots between ^15^O-water PET volume-based method and MRI ESV (**a**), EDV (**b**), SV (**c**) and LVEF (**d**). Circles (red) are values from *V*_B_ images, and squares (blue) are values from FP images. Solid lines are the mean difference, and dashed lines are the limits of agreement. Outliers were removed

Patient characteristics are seen in Table 1. Figure 1 shows an example of short-axis parametric ^15^O-water left and right ventricular blood volume, transmural MBF (MBFt = MBF multiplied with PTF) and PTF images, as well as MRI images from one patient. The top row displays images from the end-diastolic phase, and the bottom row displays images from the end-systolic phase. As can be clearly seen in the images, the thinner myocardial wall in the end-diastolic phase face results in increased partial volume effects and hence lowers apparent MBFt values. Figure 2 shows a fusion between short-axis parametric ^15^O-water blood volume images and MRI for end-systolic and end-diastolic phases. In four patients, there were significant underestimations of *V*_B_ due to a loss in counts in gated images. From here on, they are treated as outliers, and the results will be presented with and without them. This is explained in detail in the “Discussion” section. Table 2 shows correlations between PET-derived LV volumes and LVEF and MRI-derived LV volumes and LVEF including outliers. Significant correlations with MRI-based ESV, EDV, SV and LVEF were found using both *V*_B_ and FP images for surface-, volume- and count-based methods. In Table 3, correlations for the same parameters are seen but with outliers removed, resulting in higher correlations for all parameters using *V*_B_ images. Removing outliers only resulted in minor changes of correlations for all parameters when the FP method was used, except for SV where correlation decreased. Correlation plots for the volume-based method excluding outliers are shown in Fig. 3, and Fig. 4 shows corresponding Bland Altman plots. Average values of LV volumes and LVEF derived from PET and MRI are seen in Table 4 including outliers. Using *V*_B_ images, all surface-based parameters except ESVs differed significantly from MRI, whereas none of the volume-based parameters showed significant differences. For the FP method, on the other hand, all surface-based parameters differed significantly from MRI, as did the volume-based parameters SVv and LVEFv, whereas no significant differences were found for ESVv and EDVv.

**Table 1:** Patient characteristics including outliers

Age (years)	58 ± 13
Gender (female/male)	4/12
Height (cm)	176 ± 8
Weight (kg)	76 ± 10
BSA (m^2^)	1.9 ± 0.15
Heart rate (mean/range)	62/47–105
LVESV (mL/range)	90 ± 29/44–148
LVEDV (mL/range)	244 ± 65/153–412
LVSV (mL/range)	154 ± 42/109–277
LVEF (%/range)	63 ± 6/53–75
Diabetes (*n*)	0
NYHA class 1 (*n*)	15
NYHA class 2 (*n*)	1
Mitral insufficiency (*n*)	13
Aortic insufficiency (*n*)	3
CAD (*n*)	0
History of MI (*n*)	0
Hypertension (*n*)	7
History of AF (*n*)	0
Treatment	
ASA (*n*)	1
ACE/ARB (*n*)	8
CCB (*n*)	1
Diuretics (*n*)	1
Beta blockers (*n*)	3
History of PCI (*n*)	0

**Table 2:** Including outliers, correlation between PET and MRI for surface- (s), count- (c) and volume-based (v) methods. The count-based method does not provide ESV and EDV values

	Slope, *V*_B_	Intercept, *V*_B_	*r*, *V*_B_	*p*, *V*_B_
ESVs	0.93	12.7	0.73	0.001
EDVs	0.60	59.3	0.66	0.006
SVs	0.37	53.2	0.58	0.017
LVEFs	0.66	12.9	0.58	0.018
ESVv	1.00	− 3.5	0.79	< 0.001
EDVv	0.70	80.4	0.66	0.006
SVv	0.53	82.3	0.54	0.030
LVEFv	0.88	10.6	0.65	0.006
LVEFc	0.83	22.1	0.62	0.011
	Slope, FP	Intercept, FP	*r*, FP	*p*, FP
ESVs	1.20	− 8.3	0.90	< 0.001
EDVs	0.79	1.10	0.87	< 0.001
SVs	0.50	16.30	0.74	< 0.001
LVEFs	0.77	0.08	0.63	0.008
ESVv	1.32	− 19.50	0.87	< 0.001
EDVv	0.91	12.56	0.87	< 0.001
SVv	0.70	28.54	0.73	< 0.001
LVEFv	1.03	− 6.83	0.64	0.006
LVEFc	1.12	− 1.83	0.66	0.005

**Table 3:** Excluding outliers, correlation between PET and MRI for surface- (s), count- (c), and volume-based (v) methods. The count-based method does not provide ESV and EDV values

	Slope, *V*_B_	Intercept, *V*_B_	*r*, *V*_B_	*p*, *V*_B_
ESVs	1.08	10.3	0.88	< 0.001
EDVs	0.96	− 4.2	0.85	0.001
SVs	0.68	16.2	0.73	0.007
LVEFs	0.77	4.6	0.71	0.009
ESVv	1.15	− 6.2	0.89	< 0.001
EDVv	1.1	10	0.85	< 0.001
SVv	1.04	19.9	0.74	0.006
LVEFv	0.93	6.5	0.72	0.008
LVEFc	0.88	17.5	0.70	0.01
	Slope, FP	Intercept, FP	*r*, FP	*p*, FP
ESVs	1.24	− 6.2	0.95	< 0.001
EDVs	0.92	− 20.8	0.88	< 0.001
SVs	0.50	20.0	0.56	0.022
LVEFs	0.83	3.6	0.68	0.003
ESVv	1.40	− 21.6	0.89	< 0.001
EDVv	1.07	− 13.2	0.87	< 0.001
SVv	0.75	28.1	0.57	0.018
LVEFv	1.07	− 8.6	0.65	0.005
LVEFc	0.91	11.6	0.60	0.012

**Table 4:** Including outliers, average LV volumes and LVEF calculated from PET using surface- (s), count- (c), and volume-based (v) methods compared with average values from MRI

	PET, *V*_B_	MRI	Relative difference (%), *V*_B_	*p*, *V*_B_
ESVs, mL	96 ± 36	90 ± 29	8.5 ± 29.8	0.347
EDVs, mL	206 ± 60	243 ± 65	− 14.4 ± 18.4	0.012
SVs, mL	110 ± 27	154 ± 42	− 26.7 ± 15.1	< 0.001
LVEFs, %	55 ± 6	63 ± 6	− 13.8 ± 8.7	< 0.001
ESVv, mL	87 ± 36	90 ± 29	− 2.7 ± 26.8	0.622
EDVv, mL	250 ± 69	244 ± 65	4.0 ± 21.6	0.652
SVv, mL	163 ± 41	154 ± 42	8.1 ± 23.5	0.372
LVEFv, %	66 ± 8	63 ± 6	4.2 ± 9.4	0.10
LVEFc, %	75 ± 8	63 ± 6	17.8 ± 10	< 0.001
	PET, FP	MRI	Relative difference (%), FP	*p*, FP
ESVs, mL	100 ± 38	90 ± 29	11.2 ± 19.5	0.041
EDVs, mL	192 ± 59	243 ± 65	− 21.2 ± 12.7	< 0.001
SVs, mL	93 ± 28	154 ± 42	− 39.2 ± 13.6	< 0.001
LVEFs, %	49 ± 7	63 ± 6	− 23.0 ± 8.9	< 0.001
ESVv, mL	99 ± 43	90 ± 29	9.2 ± 22.5	0.126
EDVv, mL	235 ± 68	244 ± 65	− 3.5 ± 14.7	0.320
SVv, mL	136 ± 40	154 ± 42	− 10.9 ± 19.8	0.031
LVEFv, %	59 ± 9	63 ± 6	− 7.8 ± 11.4	0.017
LVEFc, %	69 ± 10	63 ± 6	8.6 ± 11.5	0.008

Results excluding outliers are shown in Table 5. Using *V*_B_ images, all surface-based parameters except EDVs differed significantly from MRI, as did EDVv and SVv which were significantly overestimated. Using FP images, all surface-based parameters were significantly different from MRI, whereas no significant difference was found for any volume-based parameter.

**Table 5:** Excluding outliers, average LV volumes and LVEF calculated from PET using surface- (s), count- (c) and volume-based (v) methods compared with average values from MRI

	PET, *V*_B_	MRI	Relative difference (%), *V*_B_	*p*, *V*_B_
ESVs, mL	103 ± 34	86 ± 28	21.3 ± 20.6	0.004
EDVs, mL	219 ± 53	232 ± 47	− 5.8 ± 11.3	0.137
SVs, mL	116 ± 24	146 ± 26	− 20.5 ± 11.6	< 0.001
LVEFs, %	54 ± 7	64 ± 2	− 15.5 ± 7.6	< 0.001
ESVv, mL	92 ± 36	86 ± 28	7.6 ± 20.6	0.221
EDVv, mL	264 ± 61	232 ± 47	14.1 ± 13.6	0.005
SVv, mL	172 ± 37	146 ± 26	17.9 ± 17.6	0.004
LVEFv, %	66 ± 8	64 ± 2	3.3 ± 9.0	0.203
LVEFc, %	74 ± 8	64 ± 2	15.8 ± 9.1	< 0.001
	PET, FP	MRI	Relative difference (%), FP	*p*, FP
ESVs, mL	100 ± 36	86 ± 28	16.9 ± 13.8	0.003
EDVs, mL	193 ± 49	232 ± 47	− 16.9 ± 10.4	< 0.001
SVs, mL	94 ± 23	146 ± 26	− 35.7 ± 13.8	< 0.001
LVEFs, %	49 ± 8	64 ± 2	− 23.0 ± 9.0	< 0.001
ESVv, mL	98 ± 43	86 ± 28	13.6 ± 21.2	0.076
EDVv, mL	236 ± 56	232 ± 47	1.5 ± 12.1	0.652
SVv, mL	137 ± 34	146 ± 26	− 5.5 ± 19.9	0.307
LVEFv, %	59 ± 10	64 ± 2	− 7.2 ± 12.5	0.074
LVEFc, %	70 ± 9	64 ± 2	9.8 ± 11.9	0.015

Excluding the outliers, surface-based LVEF was significantly underestimated for both *V*_B_ and FP images, although much less so for *V*_B_ images, whereas volume-based LVEF did not differ significantly from MRI-based values for either *V*_B_ or FP images. Count-based ejection fraction LVEFc showed a significant overestimation for both *V*_B_ and FP images, regardless of the inclusion of exclusion of outliers.

In Table 6, inter-operator repeatability is shown. Including outliers, inter-operator repeatability was good for LVEFv (ICC = 0.78, *p* < 0.001) and excellent for all other parameters (ICC > 0.80, *p* < 0.001). Excluding outliers, inter-operator repeatability was excellent for all parameters (ICC > 0.86, *p* < 0.001).

**Table 6:** Intraclass correlation coefficient (ICC) for inter-operator repeatability including and excluding outliers (*p* < 0.001 for all)

	ICC including outliers	ICC excluding outliers
ESVs	0.97	0.98
EDVs	0.99	0.99
SVs	0.95	0.94
LVEFs	0.85	0.89
ESVv	0.94	0.94
EDVv	0.99	0.99
SVv	0.93	0.92
LVEFv	0.78	0.86
LVEFc	0.80	0.89

## Discussion

In this study, the accuracy of LV volumes and LVEF derived from a single ^15^O-water PET/CT scan using cardiac-gated parametric blood volume images was assessed. High correlations between LV volumes and LVEF based on ^15^O-water parametric blood volume images towards MRI were found, despite the large number of steps required for our method. There were no significant differences between surface-, count- and volume-based methods. Agreement between PET and MRI was best for the volume-based method with no significant bias for ESV and LVEF, but with an overestimation of values for EDV and SV. For the surface-based method, a significant bias was found for ESV, SV and LVEF and no significant bias for EDV. The presence of one or two data points with high ESV, SV and LVEF show a relatively large positive difference between PET and MRI, giving the impression of a positive trend in the Bland Altman plots (Fig. 4). However, regression analysis of all Bland Altman plots gave a slope that was not significantly different from zero.

Input functions were based on cluster analysis of non-gated data, as the high statistical noise ruled out cluster analysis in single gated images. Four patients in this study had blood volume fractions of approximately 0.8 at the centre of the cavity, where it is supposed to be close to 1.0. When performing dynamic cardiac-gated acquisitions, the cardiac rebinning procedure excludes counts that originate from a cardiac cycle that ends up in between two frames of the dynamic scan. This will decrease the amplitude of the time-activity curves (TAC) in each voxel and result in an underestimation of the radioactivity concentrations especially during the first 5-s short frames, where one or two missed cycles will lead to a considerable reduction in counts compared to the non-gated input functions. In the modelling procedures, this will in turn lead to lowered blood volume fractions when cycles are missed during the first pass. Also, erroneously high PTF values will be assigned to voxels inside the cavity since the model will not be able to accurately describe the TAC of the left ventricle when the blood curve is underestimated during the first pass, which in turn will lead to erroneous estimation of volumes. This occurred in four patients in which the resulting volumetrics were generally poor when compared to MRI, and these patients were considered as outliers. This is a shortcoming of our current implementation of the cardiac rebinning procedure, and correction factors for the actual time contained in each frame should be addressed in future work. With the current implementation, it would be advisable to use a cutoff of the blood volume fraction (e.g. 0.9) below which LV volume and LVEF calculations should not be performed on parametric blood volume images. A possible modification of the data processing method that could avoid outliers as those found in the present work would be the definition of frames in terms of number of cardiac cycles instead of fixed durations in seconds. It was not possible to do this analysis with the current data, but this possibility will be investigated in future studies.

The use of gated first pass images is a more straightforward method compared to the construction of gated parametric *V*_B_ images. There are no modelling errors due to loss of counts from gated image reconstructions, and thus, higher correlations with MRI were found for FP images compared to for *V*_B_ images, for all parameters except for SV, when all patients were included. On the other hand, when outliers were excluded, *V*_B_ images showed higher correlations with MRI also for LVEF.

ECG-gated PET images are typically based on thresholding the inner contour of the tracer uptake in the myocardial wall, which is conceptually similar to the MRI approach. Myocardial wall uptake of tracers that are retained in proportion to MBF leads to variable cavity delineation and is known to produce errors in LV volumes and LVEF measurements in patients with chronic ischaemic heart disease [20]. Measuring cavity volumes using blood volume images effectively eliminates this error source, and equilibrium-gated blood volume imaging using planar scintigraphy or SPECT remains a clinically robust alternative to MRI [21]. The feasibility of synthetic blood volume images derived from parametric PET for LV volumes and LVEF measurements has not been shown before. However, true blood volume imaging by direct labelling of erythrocytes using inhaled ^15^O-CO for PET was shown to produce LV volumes and LVEF measurements with high accuracy [22, 23]. A recent publication [14] assessed the use of first pass ^15^O-water images, during the 15 s when the highest radioactivity concentrations were seen in the left ventricular cavity. This method showed a somewhat better correlation with MRI-based ESV, EDV, SV and LVEF values than the method suggested in our work. However, it should be noted that the range of volumes and LVEF in that study was much larger than in our data, which affects the correlation values. If only patients with MR-based LVEF > 53% were considered, as in our study, correlation using first pass images decreased to 0.40 for LVEF which is actually slightly lower than the present result.

The BPGS application showed some difficulties in the segmentation of parametric blood volume images, mostly when delineating the atrioventricular plane. Shrinking the edges of the volume for the automatic segmentation did help the system to delineate the atrioventricular plane better for some patients, but required manual adjustment, which might introduce observer bias. However, despite some difficulties in the segmentation, an excellent inter-operator repeatability was achieved for all parameters. Inter-operator repeatability was lowest for LVEF which partly could be explained by the narrow range of values.

The use of only eight gates is a drawback of this method, which tends to overestimate the end-systolic volumes in comparison with MRI. Using at least 16 gates is desirable; however, the low count statistics in the resulting images are likely to eliminate any potential benefit of using 16 gates instead of 8. An increase in injected dose might improve count statistics to a degree that would allow input curves to be more accurately derived from gated dynamic data. This would potentially decrease the risk of obtaining falsely low blood volume fractions and recover correct volumes from the outliers identified in this study. However, an injected activity of 400 MBq approaches the upper system limit regarding saturation. The use of the most recent generation of PET/CT or PET/MR scanners with a larger axial field of view and correspondingly higher sensitivity, time of flight capability and more robust counting statistics, might enable the use of a higher time resolution and higher doses.

In the present work, each image frame had to be sorted separately from the list-mode file, and then reconstructed into an eight-gate time-static image, involving the manual submission of 40 list-mode sorting or reconstruction assignments on the PET/CT reconstruction console and reconstruction of 160 image sets in total. Then, the 20 gated images were imported in a Matlab tool for resorting them into eight dynamic single cardiac-gate scans of 20 frames each, after which parametric images were calculated as described in the “Methods” section above. Aside from being very labour-intensive and time-consuming, this amount of manual processing is vulnerable to operator errors. Ideally, the scanner post-processing unit should automate the list-mode sorting, reconstruction and re-sorting into dynamic gated single cardiac-gated images, preferably using a frame timing definition that corresponds to full cardiac cycles. The software used for blood volume analysis originates from SPECT and has not been validated for synthetic PET blood volume images, but seems to provide reasonable results. If the software used for blood volume analysis would accommodate this, only end-systolic and end-diastolic single-gate dynamic images would need to be reconstructed. This would further reduce reconstruction times by 75%.

A limitation of the present work is that the patient population is limited to patients with mitral or aortic regurgitation with normal LV function. This highly specific patient population is though not likely to impact on the observed result. Segmentation of LV volumes is performed on either first pass images or parametric blood volume images, constructed from kinetic modelling, that are not affected by mitral or aortic regurgitation. Forward stroke volume or forward ejection fraction would have been affected but this was not a part of the present study. However, a more comprehensive assessment of the use of gated parametric ^15^O-water PET images in measuring LV volumes and LVEF is required in patients with reduced LV function. LVEF cutoff used for a decision on defibrillator implants is 35%, which is lower than in the present study, and another study will have to be done to qualify ^15^O-water in that range.

## Conclusion

Calculation of LV volumes and LVEF from gated parametric blood volume and first pass images derived from dynamic ^15^O-water PET is feasible and shows good correlation with MRI. This will enable calculation of MBF, as well as LV volumes and LVEF from a single 6-min dynamic ^15^O-water investigation, although more automated reconstruction methods are desirable for more widespread clinical use.

## Abbreviations

ECG: Electrocardiographic gating
EDV: End-diastolic volume
ESV: End-systolic volume
LVEF: Left ventricular ejection fraction
MBF: Myocardial blood flow
SPECT: Single-photon emission tomography
BPGS: Blood-pool gated SPECT
PTF: Perfusable tissue fraction
SV: Stroke volume
